# Using single-step genome-wide association analyses to compare predicted negative energy balance and a novel energy deficiency score in early-lactation Holstein cows

**DOI:** 10.3168/jdsc.2025-0778

**Published:** 2025-07-30

**Authors:** Hongqing Hu, Hadi Atashi, Sébastien Franceschini, Pauline Lemal, Clément Grelet, Yansen Chen, Katrien Wijnrocx, Hélène Soyeurt, Nicolas Gengler

**Affiliations:** 1 TERRA Teaching and Research Center, University of Liège, Gembloux Agro-Bio Tech (ULiège-GxABT), 5030 Gembloux, Belgium; 2 Department of Animal Science, Shiraz University, 71441-13131 Shiraz, Iran; 3 Walloon Agricultural Research Center (CRA-W), 5030 Gembloux, Belgium

## Abstract

This study aimed to compare the genetic architectures of logit-transformed predicted negative energy balance (LPNEB) and a novel logit-transformed energy deficiency score (LEDS) as 2 mid-infrared–derived proxies of negative energy balance in early-lactation dairy cows. A total of 30,634 records from 25,287 first-parity Holstein cows across 508 herds distributed in Walloon region of Belgium were analyzed. Genotypic data of 566,170 SNPs were available for 3,757 animals. Single-step GWAS, combined with a 50-SNP sliding window approach, was employed to explore the genetic architectures of LPNEB and LEDS. The top 10 genomic regions for LPNEB and LEDS were identified across multiple chromosomes, with 3 shared regions (BTA 1, 5, and 16). Despite these overlaps, each trait exhibited unique loci, supporting distinct genetic architectures. Positional candidate gene analyses identified 17 genes for LPNEB and 10 for LEDS, with 6 being in common. Gene Ontology enrichment analyses were then performed to explore their biological functions, although LPNEB was primarily associated with energy metabolism regulation and metabolic adaptation, whereas LEDS integrated neuronal signaling into energy homeostasis. The QTL enrichment highlighted significant associations with fertility and SCS, reinforcing a genetic basis for energy balance. These findings improve our understanding of the genetic background of LPNEB and LEDS, thereby providing new insights into the mechanisms underlying energy balance in dairy cattle.

## Highlights

• Two MIR-predicted NEB proxies (LPNEB and LEDS) were compared in Holstein cows.
• Both LPNEB and LEDS exhibit polygenic architectures.
• This study provides new insights into the mechanisms underlying energy balance.

## Graphical Abstract

**Figure:**
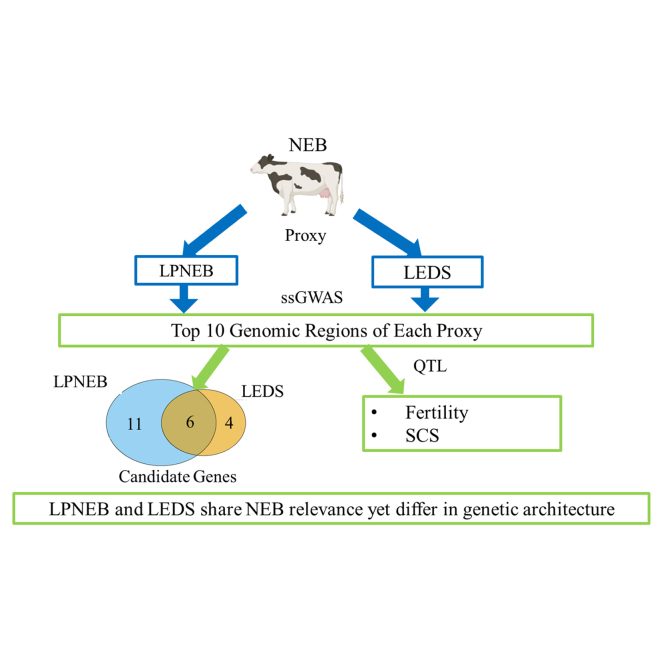
**Summary:** This study compared the genetic architectures of 2 mid-infrared (MIR)-predicted proxies of negative energy balance (NEB) in early-lactation Holstein cows: the logit-transformed predicted NEB (LPNEB) and the logittransformed energy deficiency score (LEDS). Using single-step genome-wide association study (ssGWAS) and functional enrichment analysis, the results revealed both overlapping and distinct genomic regions, biological pathways, and candidate genes for the 2 traits. These findings improve our understanding of the genetic background of LPNEB and LEDS, thereby providing new insights into the mechanisms underlying energy balance in dairy cattle. QTL = quantitative trait loci; SCS = somatic cell score.

Negative energy balance (**NEB**) is a well-recognized metabolic challenge in early-lactation dairy cows, occurring when energy intake is insufficient to meet the high demands of maintenance and milk production (Churakov et al., 2021). Prolonged NEB is associated with metabolic disorders such as ketosis and fatty liver (Esposito et al., 2014) and is linked to reduced reproductive performance, milk yield, and immune functions (Hammon et al., 2006; Pérez-Báez et al., 2019; Wathes et al., 2007). These consequences not only have an impact on cow health and welfare, but also impose substantial economic costs on dairy farms (Gohary et al., 2016; Miglior et al., 2017). Therefore, effective monitoring of NEB is crucial for maintaining herd health and productivity. Direct measurement of NEB on a large scale is challenging due to its high cost (Zachut et al., 2020). Mid-infrared (**MIR**) spectral analysis can be considered as an efficient approach to predict NEB status in dairy cows; however, the coefficients of determination of the different prediction models vary across studies (from 0.48 to 0.78; Grelet et al., 2017; Smith et al., 2019).

A promising alternative is the energy deficiency score (**EDS**), derived from MIR data, which provides a noninvasive and cost-effective method for assessing energy status (Franceschini et al., 2022). The EDS integrates multiple metabolic markers, making it a more reliable indicator of NEB than individual biomarkers. Cows in the EDS group show lower BWt and DMI, along with higher BHB and acetone in milk (Franceschini et al., 2022). These changes suggest increased fat mobilization, a key sign of NEB induced metabolic stress. The genetic correlations with blood biomarkers further support this finding, with logit-transformed EDS (**LEDS**) being strongly positively correlated with logit-transformed predicted NEB (**LPNEB**; 0.85), blood BHB (0.69), and blood nonesterified fatty acid (0.79), while showing a negative correlation with blood glucose (−0.62; Hu et al., 2025). Although both LPNEB and LEDS are MIR-based proxies for the NEB, LEDS may offer a more integrative and scalable alternative to LPNEB for genetic and management applications. Our recent study suggests moderate heritability for LPNEB and LEDS (Hu et al., 2025), highlighting the potential for genetic selection. Identifying genomic regions associated with LPNEB and LEDS could improve understanding of the relationship of these traits to metabolic adaptation and support their potential use in breeding strategies for better energy efficiency. Single-step GWAS (**ssGWAS**) provide a powerful approach to identify genomic regions associated with complex metabolic traits by integrating genotype and phenotype data from large populations.

To compare the genetic architectures of LPNEB and LEDS in early-lactation Holstein cows, this study (1) performed ssGWAS to identify genomic regions associated with LPNEB and LEDS, and (2) conducted comparative functional annotation of selected genomic regions.

The data used in this study were explained previously by Hu et al. (2025). Briefly, the dataset consisted of 30,634 records on 25,287 first-parity Holstein cows across 508 herds in Walloon region of Belgium. Some cows had multiple test-day records because MIR spectra and predicted traits were collected on different days during the early lactation period (DIM 5 to 50). To focus on early lactation, records were restricted to the period of 5 to 50 DIM. The MIR-based energy balance prediction equations used for trait estimation were developed and validated in previous studies (Grelet et al., 2017). The EDS was defined using an agglomerative hierarchical clustering approach based on 27 MIR-derived predictors (Franceschini et al., 2022). For novel observations, EDS was directly predicted from MIR spectral data, ensuring consistency with previously established methodologies. The predictive model for EDS was developed using partial least squares discriminant analysis. In the validation set, the model achieved an overall accuracy of 0.99, with a sensitivity of 0.95 and a specificity of 0.92 (Franceschini et al., 2024). The used pedigree included 76,340 animals, among which 3,757 had genotypic data for 566,170 SNPs. The genotype data were obtained from a subset of the dataset described in Chen et al. (2024). We computed LPNEB = log_10_[PNEB/(1 − PNEB)], where PNEB = 1 − [(PEB − PEB_minimum_)/(PEB_maximm_ − PEB_minimum_)], and LEDS = log_10_[EDS/(1 − EDS)], with PEB being the energy balance predicted by MIR spectra and PNEB being the NEB predicted by MIR spectra.

Similar to Hu et al. (2025), a univariate repeatability model was used to estimate variance components and GEBV for LPNEB and LEDS in first-parity cows. The model included fixed effects of herd × calving year, calving age (10 classes), calving month (12 classes), and DIM classes (4 levels). In addition, standardized DIM (centered and scaled to mean 0 and SD 1) and its quadratic term were included to model linear and nonlinear effects of DIM during early lactation. Random effects were the additive genetic effects, permanent environmental effects, and residual effects. The single-step GBLUP (**ssGBLUP**) approach was implemented, integrating pedigree-based (**A**-matrix) and genomic-based (**G**-matrix) relationships into a combined **H**-matrix (Aguilar et al., 2010) using default settings in BLUPF90+ software (version 2.48; Misztal et al., 2014). Variance components were estimated using average information REML implemented in the BLUPF90+ software. The GEBV were computed using the precondition conjugate gradient algorithm implemented in BLUPF90+.

Genome-wide association analysis was performed using the BLUPF90 family of programs with the ssGWAS approach. The SNP effects of LPNEB and LEDS were estimated using POSTGSF90 software (version 1.76; Aguilar et al., 2014). The formula used for estimating SNP effects was as stated in Wang et al. (2012):

$$\hat{u}=\;D\;Z_{g}^{'}{\left(\left(\;Z_{g}D\;Z_{g}^{'}\right)\right)}^{-1}\hat{a},$$

where **û** is the vector of SNP effects; **D** is the weight matrix of SNPs (**D** = **I**), which means the weight for all SNPs is 1; **Z***_g_* is an incidence matrix of genotypes for each SNP; and **â** is a vector of GEBV for each trait of genotyped animals. The variance of *i*th SNP is as follows: $var\left({\hat{u}}_{i}\right)=u_{i}^{2}\cdot 2p_{i}\;\left(1-p_{i}\right),$ where $u_{i}^{2}$ is the square of *i*th SNP effect and p*_i_* is the frequency of allele B at SNP *i*. A window-based approach was used to account for the combined effects of adjacent SNPs and local linkage disequilibrium structure, thereby improving the estimation of regional genetic variance. The results were presented by the proportion of additive genetic variance explained by each window of 50 adjacent SNPs with an average size of ∼240 kb. We used 1 SNP as the moving step of the window, which ensured that we would not miss genomic regions potentially associated with the trait due to the combination of SNPs. The relative total additive genetic variance of each window was computed as var(**Z***_i_***û***_i_*) divided by the total additive genetic variance, where **Z***_i_* is the matrix of their SNP content for the SNPs of interest, **û***_i_* is the vector of SNP effects, all defined in the *i*th genomic region (each window combining 50 consecutive SNPs).

Positional candidate genes and QTL annotations in the top 10 genomic regions were identified using the GALLO R package (Fonseca et al., 2020). The position (coordinate) of selected genomic regions on reference genome assembly UMD3.1 (the used chip version) was converted to the new position (coordinate) on the new reference genome assembly ARS-UCD1.2 through the Lift Genome Annotations tool (https://genome.ucsc.edu/cgi-bin/hgLiftOver). Gene Ontology (**GO**) analyses of the positional candidate genes for each trait were conducted using the g:Profiler website (Kolberg et al., 2023). The selected genomic regions identified for each trait were annotated with Cattle QTLdb (https://www.animalgenome.org/cgi-bin/QTLdb/BT/index; Hu et al., 2019). At present, Cattle QTLdb has 192,336 QTL, which were divided into 6 classes, including exterior, production, health, reproduction, milk, and meat and carcass (https://www.animalgenome.org/cgi-bin/QTLdb/BT/ontrait?class_ID=1). To avoid the deviation caused by the annotation richness of the different traits, the hypergeometric test approach was adopted for enrichment analysis (Fonseca et al., 2020). In all enrichment analyses (GO and QTL), the Benjamini–Hochberg method was used to correct multiple testing (false discovery rate <0.05). It should be noted that the Cattle QTL dataset currently does not include NEB. All statistical analyses were performed using R (version 4.2.1; R Foundation for Statistical Computing).

Figure 1 illustrates the distribution of genetic variance explained (%) across the genome for LPNEB and LEDS. Both traits conform to a polygenic inheritance model, with genetic variance contributed by numerous loci distributed across multiple chromosomes. Neither trait is controlled by a single major locus, as the top regions each explain less than 0.5% of the total additive genetic variance, indicating a polygenic genetic architecture. Table 1 compares the top 10 genomic regions explaining the largest proportion of genetic variance for LPNEB and LEDS, highlighting both shared and distinct genetic loci. Several regions, such as those on BTA 1, 5, and 16, contribute to both traits, suggesting a common genetic basis. *Bos taurus* autosomes 5 and 16 were identified in genomic regions with important effects on energy balance (Tetens et al., 2013; Krattenmacher et al., 2019). Additionally, unique genomic regions are associated with each trait, with LPNEB showing specific regions on BTA 6, 13, and 25, and LEDS exhibiting distinct regions on BTA 7, 9, and 17. These differences indicate potential variations in the genetic architecture of the 2 traits. However, we recently reported the overall genetic correlation of 0.85 between LPNEB and LEDS (Hu et al., 2025), suggesting that the common genetic basis between the 2 traits may be dispersed across other genomic regions. Additionally, the 10 biggest genomic regions selected for LPNEB and LEDS only explained a total genetic variance of 1.82% and 1.92%, respectively.

**Figure 1:**
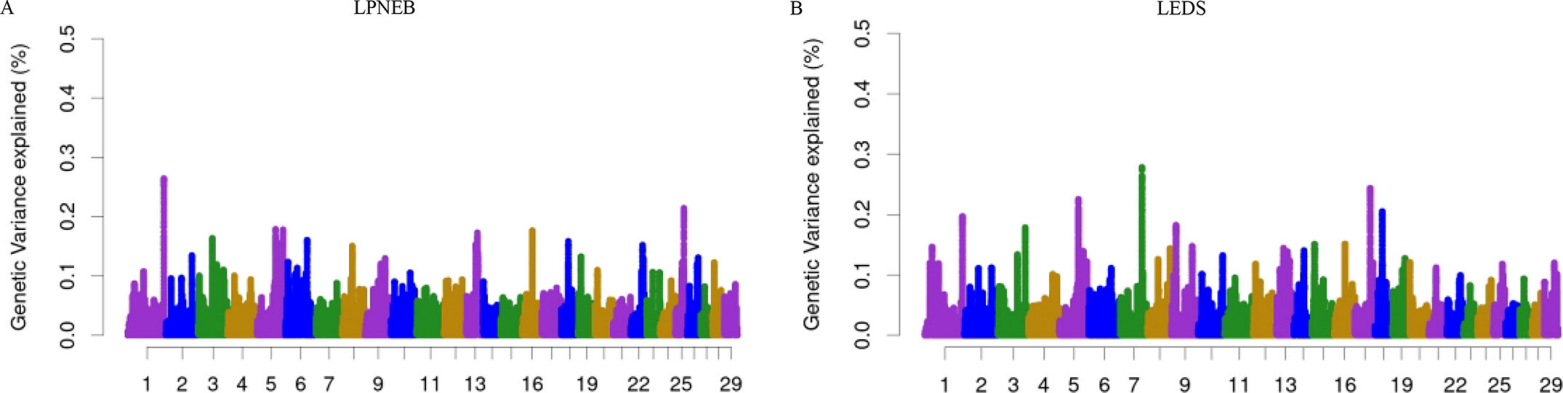
Additive genetic variance explained by windows of 50 adjacent SNPs across chromosomes for logit-transformed predicted negative energy balance (LPNEB, A) and logit-transformed energy deficiency score (LEDS, B) in early-lactation Holstein cows.

**Table 1:** Annotated genes within the top 10 genomic regions explaining the highest proportion of total genetic variance for logit-transformed predicted negative energy balance (LPNEB) and logit-transformed energy deficiency score (LEDS)^1^

Trait	BTA	Position (bp)^2^	Var^3^	Gene^4^
LPNEB	**1**	**150,974,434**–**1,510,935,985**	**0.26**	***PIGP, TTC3***
	3	55,153,255–55,383,999	0.16	*KYAT3, GTF2B, PKN2*
	**5**	**82,540,690**–**82,638,650**	0.18	***PPFIBP1***
	5	101,175,264–101,308,905	0.16	
	5	111,857,928–112,120,252	0.18	*FAM83F, TNRC6B*
	6	86,686,814–87,001,138	0.16	*ENSBTAG00000015047, UGT2A1, SULT1B1, ENSBTAG00000038214*
	13	53,637,893–53,759,622	0.17	*SIRPA*
	**16**	**44,046,234**–**44,165,527**	**0.18**	***PGD, ENSBTAG00000048790, ENSBTAG00000048747, ENSBTAG00000054239***
	18	25,753,024–25,847,046	0.16	
	25	30,079,410–30,209,679	0.21	
				
LEDS	**1**	**151,023,504**–**151,147,932**	**0.20**	***TTC3***
	3	109,338,317–109,464,393	0.18	*GRIK3*
	**5**	**82,540,690**–**82,638,650**	**0.23**	***PPFIBP1***
	7	88,653,706–88,732,303	0.28	
	9	18,311,181–18,423,364	0.18	
	9	84,820,057–84,931,668	0.15	
	15	10,352,544–10,716,253	0.15	
	**16**	**44,037,583**–**44,158,583**	**0.15**	***PGD, ENSBTAG00000048790, ENSBTAG00000048747, ENSBTAG00000054239***
	17	58,683,435–58,772,056	0.24	
	18	25,580,320–25,699,738	0.21	*CCDC102A, ADGRG5, ADGRG1*

1 Overlapping genomic regions between LPNEB and LEDS are shown in bold.

2 Position (bp) = Starting and ending coordinates of the genomic region in the UMD3.1 assembly.

3 Var = percentage of genetic variance explained by the genomic regions within the genomic region.

4 The EBSEMBL symbols of annotated genes using the *Bos taurus* ARS-UCD 1.2 assembly (http://ftp.ensembl.org/pub/release-110/gtf/bos_taurus/).

The genes annotation of the selected top 10 genomic regions for LPNEB and LEDS identified a total of 17 positional candidate genes for LPNEB and 10 positional candidate genes for LEDS, with 6 positional candidate genes shared between the 2 traits. The GO enrichment analysis for LPNEB and LEDS indicated a notable overlap in metabolic processes (Figure 2A and 2B), particularly in d-gluconate metabolic (GO: 0019521), d-gluconate catabolic (GO: 0046177) and pentose biosynthesis (GO: 0019322), all influenced by the *PGD* gene, suggesting common regulatory mechanisms for energy balance. However, LPNEB is more strongly associated with transferase activity (GO: 0016740; *TTC3*, *KYAT3*, *GTF2B*, *PKN2*, *UGT2A1*, *SULT1B1*, *ENSBTAG00000038214*), indicating a broader metabolic adaptation role. In contrast, LEDS exhibits additional enrichment in neuronal signaling pathways (GO: 0097451, *ADGRG1*; GO: 0097449, *ADGRG1*), suggesting a potential link between metabolic and neurological regulation. These results suggest a shared genetic basis for energy balance, with LPNEB emphasizing metabolic detoxification and enzymatic regulation, and LEDS integrating neuronal signaling into energy homeostasis.

**Figure 2:**
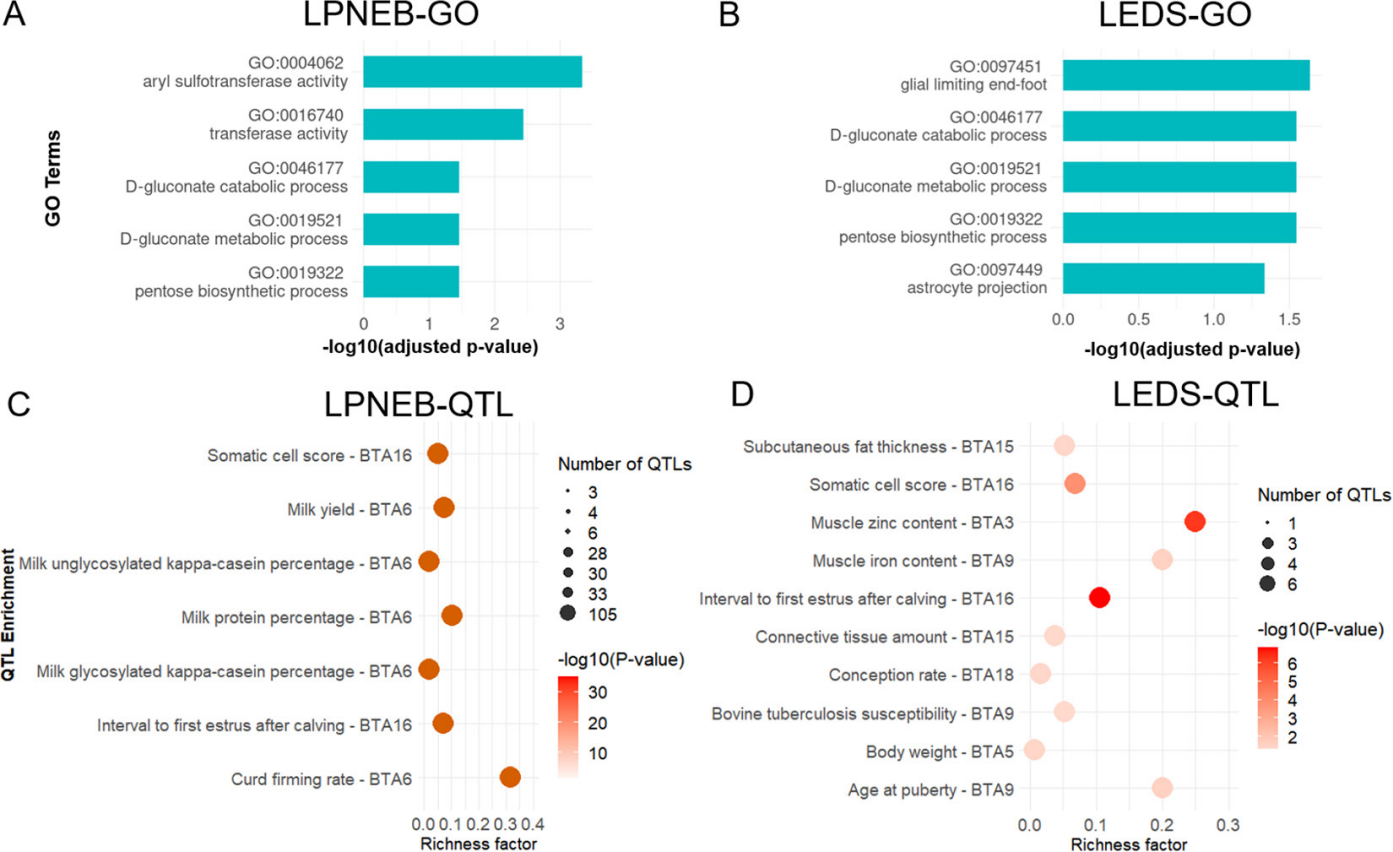
The Gene Ontology (GO) terms (A and B) and QTL annotation (C and D) based on candidate genes of logit-transformed predicted negative energy balance (LPNEB) and logit-transformed energy deficit score (LEDS).

The QTL enrichment analysis of the top 10 genomic regions associated with LPNEB and LEDS against the Cattle QTLdb identified substantial associations with multiple economically important traits (Figure 2C and 2D). The QTL enrichment analyses of LPNEB and LEDS revealed 2 shared enriched regions: SCS and interval to first estrus after calving, both on BTA16. These suggest energy metabolic potential has common regulatory mechanisms related to health and fertility (Cole et al., 2011). Beyond these overlaps, LPNEB showed strong enrichment on BTA6 for milk production traits, including milk yield, protein percentage, and kappa-casein content. Previous studies found milk production–related QTL within this genomic region (Buitenhuis et al., 2016; van den Berg et al., 2020; Pedrosa et al., 2021), suggesting a potential genetic link between energy balance and milk composition. In contrast, LEDS was enriched for traits related to muscle development, fat metabolism, immunity, and growth, distributed across multiple chromosomes, such as BTA3, BTA5, BTA9, BTA15, and BTA18. The association with muscle zinc content is particularly noteworthy given its role in protein synthesis, immune function, and overall metabolic efficiency (Hawken et al., 2012; Mateescu et al., 2017). These findings indicate that LPNEB and LEDS share QTL but are also influenced by distinct genetic architectures.

In conclusion, we found that both LPNEB and LEDS exhibited polygenic architectures, but they did not have the same magnitude of genetic variance explained. The presence of shared genomic regions was found to partially explain the high genetic correlation observed between LPNEB and LEDS. However, a more detailed interpretation requires the assessment of local genetic correlations across the genome. Annotation of candidate genes and their functional analysis showed that LPNEB and LEDS were indeed related to energy metabolism in dairy cows. The findings of these QTL analyses indicated that both LPNEB and LEDS are genetically associated with fertility, and immune response (SCS), reinforcing their relevance in cattle breeding for improving production and health outcomes. These findings improve our understanding of the genetic background of LPNEB and LEDS, thereby providing new insights into the mechanisms underlying energy balance in dairy cattle.
